# Antimicrobial Resistance to WHO-Categorised Critically Important Antimicrobials in Piglet-Derived *Escherichia coli* and Enterococci: A One Health Approach

**DOI:** 10.3390/microorganisms14102342

**Published:** 2026-10-14

**Authors:** Maria Manuel Donato, Olga Cardoso, Sara Carolina Henriques, Fernando Ramos

**Affiliations:** 1 CIMAGO, Faculdade de Medicina, Universidade de Coimbra, 3000-548 Coimbra, Portugal; 2 CERES, Department of Chemical Engineering, Faculdade de Ciências e Tecnologia, Universidade de Coimbra, 3030-790 Coimbra, Portugal; ocardoso@ci.uc.pt; 3 Faculdade de Farmácia, Universidade de Coimbra, 3000-548 Coimbra, Portugal; framos@ff.uc.pt; 4 Research Institute for Medicines (iMed.ULisboa), Faculty of Pharmacy, Universidade de Lisboa, 1649-003 Lisbon, Portugal; sarachenriques@campus.ul.pt; 5 LAQV REQUIMTE, 4050-313 Porto, Portugal

**Keywords:** WHO-categorised critically important antimicrobials, antimicrobial resistance, *Escherichia coli*, enterococci, piglets, multidrug resistance, One Health

## Abstract

Antimicrobial resistance (AMR) is a critical health issue affected by human, animal, and environmental factors within a One Health framework. Livestock production, particularly pig farming, contributes to the emergence and spread of resistant bacteria through antimicrobial use and selective pressure. This study characterized the antimicrobial resistance profiles of *Escherichia coli* and enterococci isolated from piglet faeces in Portugal, with emphasis on antimicrobials classified by the World Health Organization (WHO) according to their importance for human medicine. A total of 340 *E. coli* and 130 *Enterococcus* isolates were tested using disk diffusion, and the resistance frequencies were calculated at isolate level. *E. coli* showed high resistance to commonly used antimicrobials, including amoxicillin–clavulanic acid (67.1%) and ciprofloxacin (43.2%), a highest priority critically important antimicrobial (HPCIA). Resistance was also found to human-only drugs such as piperacillin (67.4%) and, at a lower frequency, imipenem (2.6%), a last-resort carbapenem. Multidrug resistance was detected in 61.2% of isolates. Among enterococci, *E. faecium* exhibited high resistance to imipenem (64.4%), while *E. faecalis* and other *Enterococcus* species showed notable resistance to ciprofloxacin. Overall, resistance crossed all WHO antimicrobial categories. This finding highlights the potential role of pig production in the dissemination of AMR across ecosystems and suggests the need for strengthened One Health surveillance and antimicrobial stewardship efforts.

## 1. Introduction

Antimicrobial resistance (AMR) is a critical global challenge that extends beyond the boundaries of clinical and veterinary medicine, encompassing complex ecological dynamics across human, animal, and environmental systems. The World Health Organization (WHO) has identified AMR as one of the top global public health threats of the twenty-first century, with resistant infections causing substantial morbidity, mortality, and economic losses worldwide. Increasing evidence demonstrates that the emergence and dissemination of AMR are driven not only by human antimicrobial consumption but also by antimicrobial use in food-producing animals and the subsequent release of resistant microorganisms and resistance genes into the environment [1,2,3].

The interconnected nature of AMR has reinforced the adoption of the One Health framework, which recognizes that the health of humans, animals, plants, and ecosystems is intrinsically linked. Under this approach, surveillance, prevention, and control strategies must be coordinated across sectors to effectively address the transmission of resistant microorganisms and resistance determinants among humans, livestock, wildlife, and environmental compartments [1]. In this context, livestock production systems are recognized as important interfaces where antimicrobial use, bacterial selection, and environmental dissemination converge, making them a key focus of One Health AMR surveillance programmes [2,3,4].

Pig farming plays a particularly important role in European animal production and constitutes one of the sectors with historically high antimicrobial consumption. Piglets are especially relevant because they are highly susceptible to bacterial infections during the post-weaning period, often resulting in antimicrobial treatments. Consequently, antimicrobial-resistant bacteria are frequently detected in swine production systems, with the proportion of colonized animals reaching high levels in intensive production settings. Once colonized, pigs may continuously shed resistant bacteria in their faeces, facilitating their dissemination into production environments and surrounding ecosystems [4,5,6].

Manure management practices further contribute to this challenge. The application of animal manure as agricultural fertilizer facilitates the release of antimicrobial residues, resistant bacteria, and antimicrobial resistance genes into soils, groundwater, and surface waters, promoting their persistence and horizontal transfer within environmental microbiomes [1,5,6]. These processes are especially relevant from a One Health perspective because environmental compartments may act both as reservoirs and transmission pathways, allowing resistant bacteria selected in livestock production systems to re-enter human populations through water, food chains, occupational exposure, or direct animal contact [7,8,9].

Portugal has one of the most important pig production sectors in Southern Europe, with swine farming representing a significant component of the national livestock industry. Although antimicrobial use in Portuguese livestock has decreased substantially in recent years, veterinary antimicrobials continue to be widely used in intensive production systems [10]. According to data reported by the Direcção Geral de Alimentação e Veterinária (DGAV) within the European Surveillance of Veterinary Antimicrobial Consumption (ESVAC) programme, Portugal achieved an approximately 58% reduction in veterinary antimicrobial consumption between 2018 and 2022, reflecting the impact of national and European strategies promoting prudent antimicrobial use [11]. Significant reductions were also observed for critically important antimicrobials, including polymyxins, while stricter controls were implemented for fluoroquinolones and third- and fourth-generation cephalosporins. Nevertheless, the persistence of antimicrobial-resistant bacteria in livestock production systems remains a major concern, particularly because reductions in antimicrobial consumption may not immediately translate into reductions in resistance prevalence [12].

*Escherichia coli* and *Enterococcus* spp. are widely used as indicator organisms in AMR surveillance due to their abundance in faecal material, ease of isolation, and ability to acquire and disseminate mobile genetic elements. Both genera can survive for prolonged periods outside the host and have been repeatedly detected in manure-amended soils, surface waters, and sediments, where they contribute significantly to the environmental resistome. Importantly, several resistance determinants commonly identified in livestock-associated *E. coli* and *Enterococcus* isolates are also relevant in human clinical settings, highlighting their importance as sentinel organisms within integrated One Health surveillance systems [7,8,9].

Although European surveillance programmes have documented AMR trends in livestock-associated bacteria, data specifically focused on piglets remain scarce, particularly in Portugal. Therefore, the present study aimed to characterize the antimicrobial resistance profiles of *E. coli* and *Enterococcus* spp. isolated from piglet faeces using antimicrobials classified as critically important for human medicine, antimicrobials used exclusively in human medicine, and antimicrobials authorized for both human and veterinary use according to the World Health Organization (WHO) List of Medically Important Antimicrobials [13], from a One Health perspective.

## 2. Materials and Methods

### 2.1. Sample Collection, Bacterial Isolation, and Identification

Seventy-five piglet feces samples were collected from November 2018 to May 2019, in a slaughterhouse in Aveiro that receives piglets from various regions of Portugal, due to the high demand of the local restaurants serving roasted suckling pig, a gastronomic delicacy of the Aveiro region. Sample collection was performed under the supervision of a veterinarian.

The distribution of these pig farms is higher in the Portuguese central region, and the district of Leiria is the one with the highest livestock density; hence, most of the samples were from this district [n = 33 including piglets from Pombal (n = 10; five sampled in March and five in April 2019), Portela (n = 9; five in March and four in May 2019), Alcobaça (n = 5; January 2019), Batalha (n = 5; February 2019), and Caldas da Rainha (n = 4; February 2019)], followed by the Aveiro district (n = 19; five in December 2018, five in March 2019, five in April 2019, and four in May 2019) (on the border between the north and center of Portugal). Also, Coimbra (n = 5; December 2018) and Castelo Branco (n = 4; May 2019) belong to center region. Santarém (n = 5; November 2018) is in Lisboa e Vale do Tejo region, and Faro (n = 9; five in March and four in May 2019) is in Algarve, south of Portugal.

*E. coli* were isolated from a total of 75 samples, while the *Enterococcus* isolates were obtained only from 43 of the same samples.

Samples (10 g) were mixed with 90 mL of a Tryptone salt broth (Oxoid, Basingstoke, Hampshire, UK). Suspensions were transferred to sterile stomacher bags and homogenized for 2 min in a stomacher blender. Serial dilutions were made in Tryptone salt broth.

The isolation of *E. coli* was carried out using lauryl sulphate agar (37 °C, 24–48 h) (Oxoid). The yellow-coloured colonies on the lauryl sulphate agar are *E. coli*. Up to six morphologically different colonies were selected to increase the recruitment of a variety of *E. coli* strains, and were picked into peptone water, after an oxidase test negative, and incubated overnight at 37 °C. A drop of Kovacs reagent was added to each culture, and a change of the color to cherry-red identified this bacterium. If there was any doubt about identification, the API 20E (BioMérieux, Linda-a-Velha, Portugal) was used. The isolation of enterococci was done in Slanetz Bartley Agar (37 °C, 24–48 h) (Oxoid). The red coloured colonies are representative of *Enterococcus,* and for further identification, catalase, Bile Esculin Agar (Oxoid), and Trypticase Soy broth + 6.5% NaCl tests were done. If there was any doubt about the identification, the API STREP (BioMérieux) was used.

*Enterococcus* isolates were identified to species level (*E. faecalis*, *E. faecium* or *Enterococcus* spp.) by real-time PCR (LightCycler, Roche Diagnostics, Mannheim, Germany) on crude bacterial DNA, as previously described [14].

PCR was performed in a volume of 20 µL containing 4.0 µL of LightCycler FastStart DNA MasterPLUS SYBR Green I^®^ (Roche Diagnostics, Mannheim, Germany). An initial denaturation cycle at 95 °C for 10 min was performed in all cases, followed by 45 amplification cycles. Specific denaturation, annealing, and extension conditions for each set of primers are shown in Table 1. Melting curves were also plotted automatically and analyzed with the LightCycler software (v.1.5). PCR products were checked on 2% agarose gels stained with ethidium bromide and visualized with UV light. By comparing both results, it was possible to establish specific melting temperatures to identify each gene; positive and negative controls were always included. To avoid methodological errors, a different technician, blinded to previous results, repeated all procedures.

### 2.2. Antimicrobial Susceptibility Testing

Antimicrobial susceptibility was assessed using the agar disk diffusion method according to EUCAST guidelines [16]. All the antimicrobial agents were from Liofilchem^®^s.r.l., Roseto degli Abruzzi, Italy.

Piperacillin—30 µg (PIP), aztreonam—30 µg (AZT), imipenem—10 µg (IP), vancomycin—5 µg (VAN), tigecycline—15 µg (TIG), linezolid—10 µg (LNZ) belong to the group of antimicrobials authorized for exclusive use in humans [13].

Cefepime—30 µg (FEP), ceftazidime—10 µg (CAZ), ciprofloxacin—5 µg (CIP), amikacin—30 µg (AK), cefoxitin—30 µg (FOX), ampicillin—10 µg (AMP), amoxicillin–clavulanic acid—20–10 µg (AMC), and trimethoprim–sulfamethoxazole—23.75–1.25 µg (SXT) belong to the group of antimicrobials authorized for human and animal use. Within this group, FEP, CAZ, and CIP are classified as Highest Priority Critically Important Antimicrobials (HPCIA); AK as Critically Important Antimicrobials (CIA); and FOX, AMP, AMC, and SXT as Highly Important Antimicrobials (HIA) [13].

*E. coli* isolates were tested against AMC, PIP, FOX, CAZ, FEP, AZT, IP, AK, CIP, and SXT.

*Enterococcus* isolates were tested against VAN, CIP, LNZ, TIG, AMP, and IP.

*E. coli* ATCC 25922 and *E. faecalis* ATCC 29211 were used as quality control strains. Antibiograms of these control strains were performed at the same time as those of the respective isolates. The results were always within the limits defined by EUCAST (2020) for these bacteria [17].

Additionally, the presence of MBLs (except KPC and OXA) was checked by IP/IP+EDTA double disk test.

Multidrug resistance (MDR) was defined as acquired resistance to three or more antimicrobial classes [18].

### 2.3. Statistical Analysis and Data Presentation

Antimicrobial resistance was summarized using descriptive statistics. Resistance frequencies were calculated at the isolate level and therefore represent the proportion of bacterial isolates resistant to each antimicrobial, rather than the proportion of piglets carrying resistant bacteria. For *E. coli* and *Enterococcus* isolates, the number and percentage of resistant isolates for each antibiotic were calculated, and 95% confidence intervals (CI) for these proportions were estimated using the Wilson method. Antibiotics were classified according to the WHO categorization of antimicrobial importance for human medicine [13].

Results are presented as heatmaps displaying the estimated resistance percentages and as forest plots showing the corresponding resistance estimates with their 95% CI.

For Enterococcus isolates, differences in resistance proportions among the three *Enterococcus* groups (*E. faecalis*, *E. faecium*, and *Enterococcus* spp.) were assessed for selected antimicrobials using Fisher’s exact test. When the overall test indicated a significant difference, pairwise comparisons were performed using Fisher’s exact test, with *p*-values adjusted for multiple comparisons using the Holm method. A significance level of α = 0.05 was used.

All statistical and graphical analyses were performed using R version 4.6.1 (R Core Team, Vienna, Austria, 2026).

## 3. Results

### 3.1. Antimicrobial Resistance in Escherichia coli

**Figure 1:**
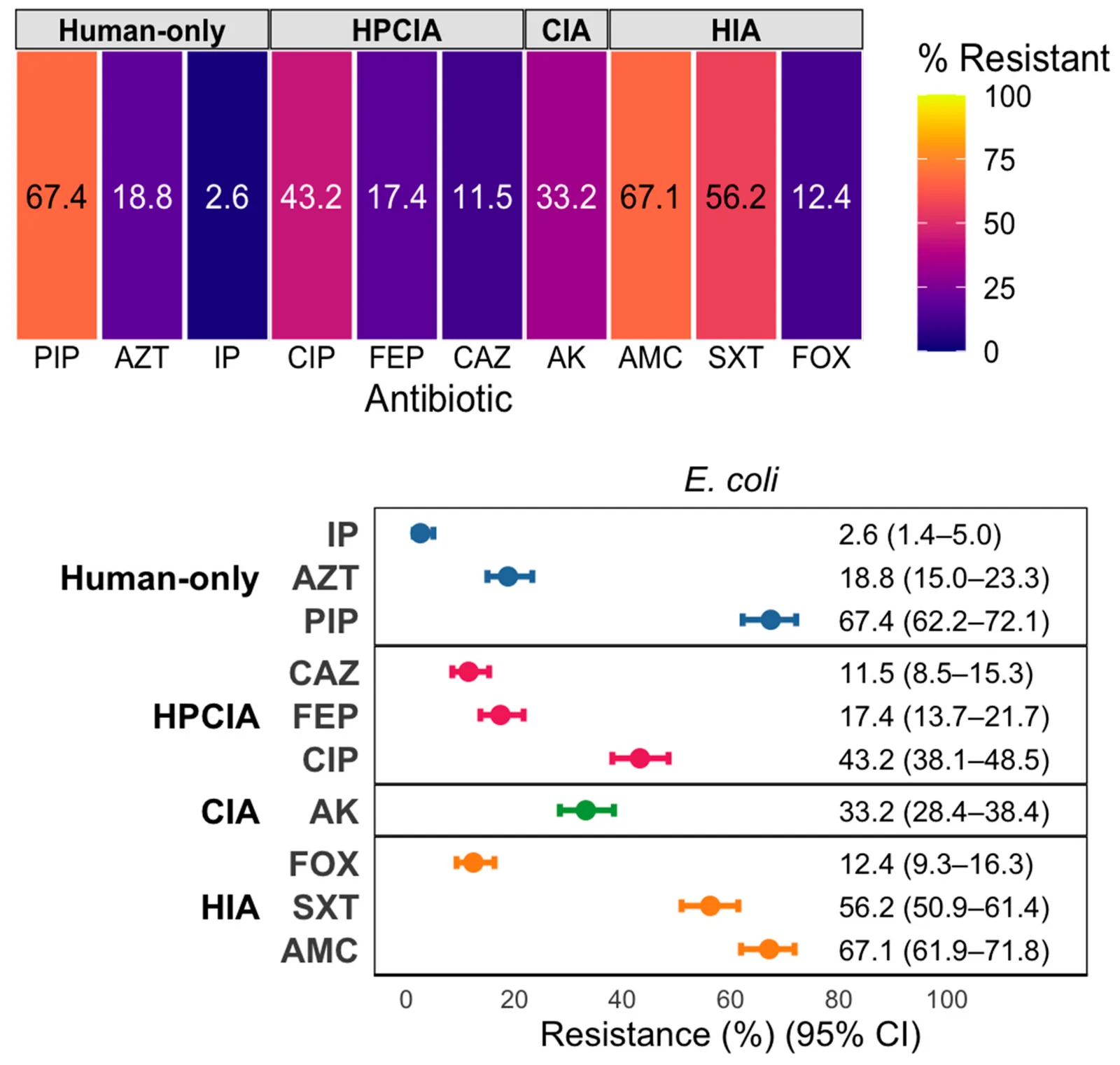
Antibiotic resistance profile of *Escherichia coli* isolates. Above: Heatmap showing the percentage of *E. coli* isolates resistant to each antibiotic tested: Piperacillin (PIP), Aztreonam (AZT), Imipenem (IP), Ciprofloxacin (CIP), Cefepime (FEP), Ceftazidime (CAZ), Amikacin (AK), Amoxicillin + Acid Clavulanic (AMC), Sulfamethoxazole + trimethoprim (SXT), Cefoxitin (FOX). Colour intensity indicates the percentage of resistant isolates, with yellow representing higher resistance and blue representing lower resistance. Antibiotics on the *x*-axis are grouped according to the WHO antimicrobial importance classification: Human-only, Highest Priority Critically Important Antimicrobials (HPCIA), Critically Important Antimicrobials (CIA), and Highly Important Antimicrobials (HIA), and are ordered from highest to lowest resistance within each group. Numerical values within each tile indicate the percentage of resistant isolates. Below: Forest plot showing the percentage of resistant isolates for each antibiotic among *E. coli.* Points represent estimated resistance percentages, and horizontal lines represent the corresponding 95% confidence intervals (CIs). Antibiotics are grouped and coloured according to the WHO antimicrobial importance classification: Human-only (blue), HPCIA (pink), CIA (green), and HIA (orange). Values shown beside each estimate represent resistance percentages with their 95% confidence intervals.

*E. coli* isolates demonstrated high resistance rates to multiple antimicrobial agents, particularly those classified by WHO as critically important for human medicine. Among the antimicrobials classified as Human-only, the observed resistance was: 67.4% (95% CI: 62.2–72.1) to PIP, an anti-pseudomonas penicillin; 18.8% (95% CI: 15.0–23.3) to AZT, a monobactam; and 2.6% (95% CI: 1.4–5.0) to IP, a carbapenem. Among the HPCIA, the observed resistance was: 43.2% (95% CI: 38.1–48.5) to CIP, a fluoroquinolone; 17.4% (95% CI: 13.7–21.7) to FEP, a fourth-generation cephalosporin, and 11.5% (95% CI: 8.5–15.3) to CAZ, a third-generation cephalosporin. The resistance observed to AK, an aminoglycoside, classified as CIA, was 33.2% (95% CI: 28.4–38.4). Among the antimicrobials classified as HIA, the observed resistance was 67.1% (95% CI: 61.9–71.8) to AMC, a beta-lactam associated with a beta-lactamase inhibitor, 56.2% (95% CI: 50.9–61.4) to SXT, an association of sulfamethoxazole + trimethoprim; and 12.4% (95% CI: 9.3–16.3) to FOX, a second-generation cephalosporin (Figure 1) (Table S1).

The phenotypic test for the detection of MBLs was negative in all the *E. coli* resistant to IP.

Overall, 208 out of 340 isolates (61.2%) met the criteria for multidrug resistance. Among these isolates, 12 were resistant to CIP (HPCIA), SXT (HIA), and beta-lactams, except IP (Human-only), with variability to AK (CIA), and one was resistant to all antibiotics used.

### 3.2. Antimicrobial Resistance in Enterococci

The 130 *Enterococcus* isolates comprised *E. faecalis* (n = 40, 30.8%), *E. faecium* (n = 45, 34.6%), and *Enterococcus* spp. (n = 45, 34.6%).

The highest resistance in *E. faecalis* was observed to CIP, an HPCIA, at 45.0% (95% CI: 30.7–60.2), followed by IP, a Human-only antimicrobial, at 15.0% (95% CI: 7.1–29.1). A similar pattern was observed for *Enterococcus* spp., with resistance of 44.4% (95% CI: 30.9–58.8) to CIP and 13.3% (95% CI: 6.3–26.2) to IP. In contrast, *E. faecium* exhibited the highest resistance to IP at 64.4% (95% CI: 49.8–76.8), followed by CIP at 15.6% (95% CI: 7.7–28.8).

**Figure 2:**
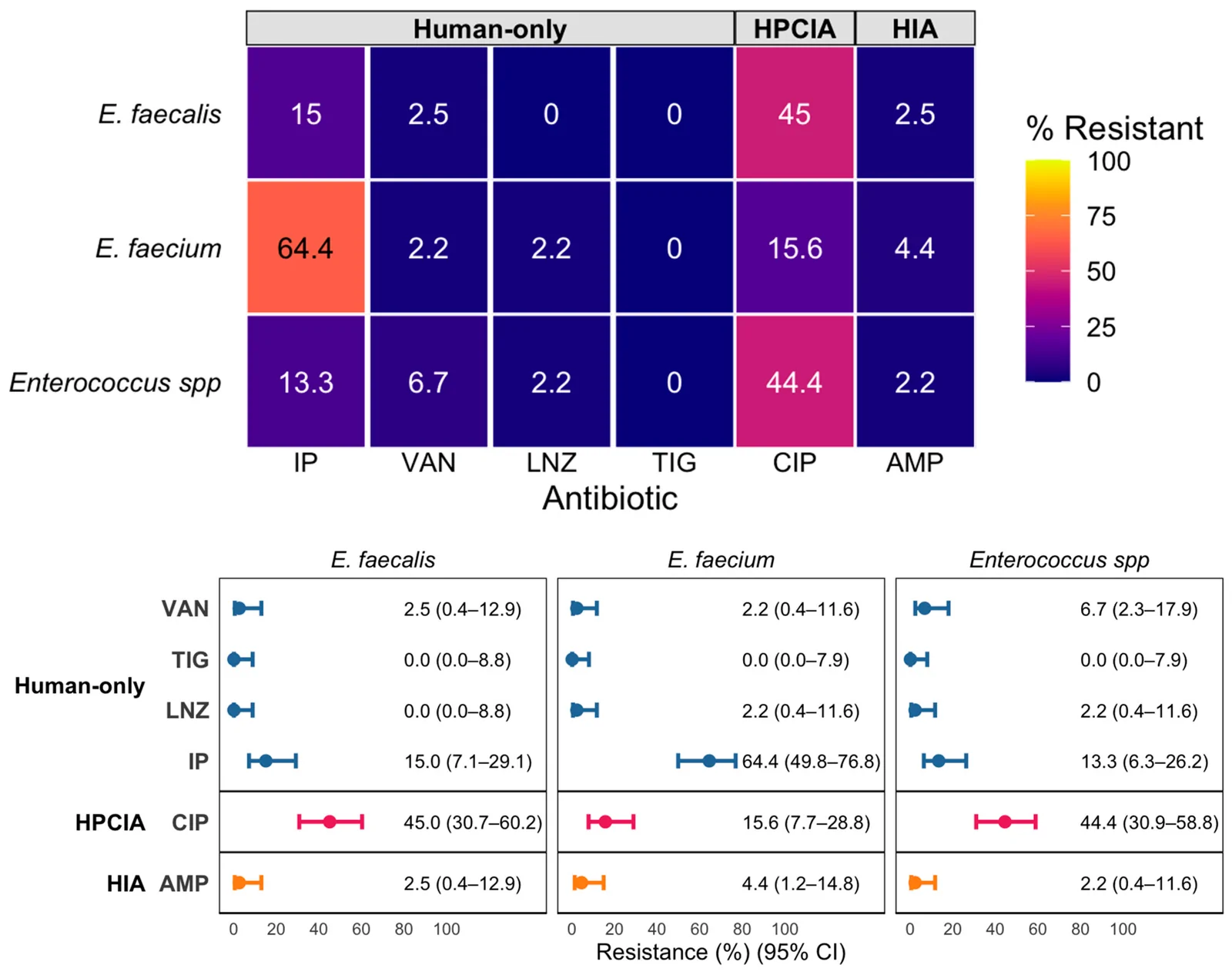
Antibiotic resistance profile of *Enterococcus* spp. Above: Heatmap showing the percentage of *E. faecalis*, *E. faecium*, and *Enterococcus* spp. isolates resistant to each tested antibiotic: Imipenem (IP), Vancomycin (VAN), Linezolid (LNZ), Tigecycline (TIG), Ciprofloxacin (CIP), Ampicillin (AMP). Colour intensity indicates the percentage of resistant isolates, with yellow representing higher resistance and blue representing lower resistance. Antibiotics on the *x*-axis are grouped according to the WHO antimicrobial importance classification: Human-only, Highest Priority Critically Important Antimicrobials (HPCIA), Critically Important Antimicrobials (CIA), and Highly Important Antimicrobials (HIA), and are ordered from highest to lowest resistance within each category. Numerical values within each tile indicate the percentage of resistant isolates. Below: Forest plot showing the percentage of resistant isolates for each antibiotic among *E. faecalis*, *E. faecium*, and *Enterococcus* spp. Points represent estimated resistance percentages, and horizontal lines represent the corresponding 95% confidence intervals. Antibiotics are grouped and coloured according to the WHO antimicrobial importance classification: Human-only (blue), HPCIA (pink), and HIA (orange). Values shown beside each estimate represent resistance percentages with their 95% confidence intervals.

Resistance to VAN and LNZ (Human-only), and to AMP (HIA), was low across all species, ranging from 0.0% to 6.7%, and none of the isolates exhibited resistance to TIG (Human-only) (Figure 2) (Table S2).

Resistance patterns differed significantly among the three enterococci groups for both CIP and IP. For CIP, resistance was significantly higher in *E. faecalis* (45.0%) and *Enterococcus* spp. (44.4%) than in *E. faecium* (15.6%) (overall Fisher’s exact test, *p* = 0.003; Holm-adjusted pairwise *p* = 0.016 for both comparisons), whereas no significant difference was observed between *E. faecalis* and *Enterococcus* spp. (*p* = 1.000). Conversely, for IP, resistance was significantly higher in *E. faecium* (64.4%) than in *E. faecalis* (15.0%) and *Enterococcus* spp. (13.3%) (overall Fisher’s exact test, *p* < 0.001; Holm-adjusted pairwise *p* < 0.001 for both comparisons), with no significant difference between *E. faecalis* and *Enterococcus* spp. (Holm-adjusted *p* = 1.000). The phenotypic test for the detection of MBLs was negative in all the enterococci resistant to IP.

*E. faecium* presented the greatest resistance to an antibiotic classified as Human-only. *E. faecalis* and *Enterococcus* spp. were highly resistant to an antibiotic classified as HPCIA.

## 4. Discussion

This study provides an integrated assessment of AMR in *E. coli* and enterococci species isolated from piglets’ faeces in Portugal, focusing on antimicrobials of high and critical importance for human medicine and contextualising resistance profiles using WHO priority classifications [13], addressing a critical knowledge gap regarding early-stage pig production within a One Health approach. Piglet faecal samples were selected as a relevant matrix within the One Health framework. The faeces originate directly from animals, are subsequently introduced into the environment through manure management practices, and may contribute to the contamination of soils and water bodies. Consequently, these samples can provide valuable information on the circulation and potential dissemination of antimicrobial-resistant bacteria across interconnected animal, environmental, and human reservoirs. The presence of clonal isolates cannot be excluded, but the geographical separation of the farms where the piglets were raised, and the diversity observed in antimicrobial resistance phenotypes of bacteria, indicate that clonality was likely limited and did not significantly affect the results or conclusions of this study. Nevertheless, irrespective of the presence of clonal isolates, piglets continuously excrete bacteria into the environment, thereby contributing to the bacterial burden in soil and water and potentially promoting environmental contamination.

Resistance frequencies in this study were calculated at the isolate level and therefore describe the resistance profile of the bacterial isolates recovered from piglet faeces rather than the prevalence of resistance among individual piglets. As multiple isolates may originate from the same fecal sample, these findings should not be interpreted as animal-level resistance prevalence.

The resistance patterns observed in *E. coli* were broadly consistent with those reported across European pig production systems and closely align with Portuguese surveillance data [8,19]. Resistance determinants to β-lactams, sulphonamides, and fluoroquinolones are frequently associated with plasmids, integrons, and other mobile genetic elements, facilitating their persistence and dissemination within bacterial populations [20,21]. The high prevalence of resistance to penicillins, particularly amoxicillin–clavulanic acid, sulphonamides, and fluoroquinolones reflects their use in veterinary medicine and the selective pressure exerted on the intestinal microbiota of pigs, and has been repeatedly documented in *E. coli* from pigs in Portugal, confirming that the patterns detected in piglets mirror established national trends [19,22,23,24].

Of particular concern was the detection of some *E. coli* isolates resistant to third- and fourth-generation cephalosporins, which the WHO classifies as HPCIA, and which have also been reported in other Portuguese studies [19,22,25]. These compounds are important therapeutic options for severe human infections, and the dissemination of resistance determinants among livestock-associated bacteria may contribute to broader public health challenges related to antimicrobial resistance. Resistance to these compounds is frequently associated with extended-spectrum β-lactamase (ESBL) and AmpC β-lactamase determinants that can spread efficiently within Enterobacterales populations [25]. Their occurrence in piglets suggests that resistant bacteria or resistance genes may establish early in life through both vertical transmission and horizontal gene transfer within dense intestinal microbial communities, environmental exposure, or contact with colonised animals [20,26].

Although resistance to IP was rare, as reported by other authors [19], its detection remains noteworthy because carbapenems are not authorised for use in food-producing animals in the European Union. The occurrence of carbapenem resistance in livestock-associated bacteria highlights the complexity of AMR ecology, whereby resistance can emerge and persist independently of direct exposure to the corresponding antimicrobial. Potential mechanisms include co-selection by other antimicrobial classes (other beta-lactams), indirect exposure to environmental reservoirs harbouring carbapenem resistance determinants, or bacterial exchange across human, animal, and environmental compartments [27]. Even when detected at low frequencies, resistance to last-resort antimicrobials is epidemiologically relevant because it may indicate the presence of resistance determinants capable of moving across ecological boundaries.

The integration of WHO antimicrobial priority classifications into the resistance heatmaps provides additional insight into public health relevance. Resistance in *E. coli* was most pronounced against Human-only last-line agents, although resistance crossed all the antimicrobial categories grouped by the WHO.

Comparable MDR levels have been reported in Portuguese and other European surveillance programmes, confirming that commensal *E. coli* in pigs can act as important reservoirs of resistant bacteria [8,22,28]. In the present study, 61.2% of the *E. coli* isolates were classified as MDR, a prevalence slightly higher than the 58.8% reported in other studies in Portugal and comparable to the levels described in Romania, Spain, and Bulgaria; in contrast, higher MDR prevalence has been reported in Italy, Cyprus, and Greece [28]. From a mechanistic perspective, MDR likely reflects the cumulative acquisition and maintenance of multiple resistance determinants within bacterial populations exposed to diverse antimicrobial and environmental selective pressures. Once established, MDR strains may experience only limited fitness costs, allowing them to persist in host populations even in the absence of sustained antimicrobial exposure. The piglet gastrointestinal tract provides favourable conditions for horizontal gene transfer because of the high bacterial densities and proximity among microbial populations [20,21]. As a result, commensal *E. coli* may function as a reservoir and exchange platform for resistance determinants that can subsequently spread to other bacterial taxa.

Resistance patterns among enterococci differed markedly by species, reflecting well-established biological and ecological differences within the genus. Elevated resistance to CIP in *E. faecalis* and non-typed *Enterococcus* spp., together with the high resistance to IP observed in *E. faecium*, is consistent with previous international and Portuguese findings [22,29,30]. Alterations in PBP5 are known to contribute to high-level resistance to both AMP and IP in *E. faecium*. Nevertheless, the isolates analyzed in this study displayed high resistance to IP while maintaining relatively low resistance to AMP. This phenotype has been reported previously, suggesting that additional factors may influence the expression of β-lactam resistance [31].

The low resistance to VAN and LNZ, observed in this study is reassuring and reflects trends reported in Portuguese and European surveillance following restrictions on the use of certain antimicrobials [8,22]. The absence of TIG resistance across all *Enterococcus* species further supports the effectiveness of restricted use policies. Nevertheless, the detection of vancomycin-resistant enterococci (VRE), even at low prevalence, is epidemiologically relevant. Sporadic VRE have previously been reported in Portuguese livestock and food matrices [22], indicating that clinically important resistance determinants can persist outside healthcare settings. The distinct resistance profiles of *E. faecalis* and *E. faecium* highlight the importance of species-level identification in AMR surveillance, as *E. faecium* is more frequently linked to hospital outbreaks and severe human infections, raising concerns about potential exchange of resistance genes at the animal–human interface [32,33]. In *E. faecium*, remarkable resistance was observed to IP, an antibiotic belonging to Human-only, being a last-resort antibiotic in a hospital setting. In *E. faecalis* and *Enterococcus* spp., resistance clustered primarily around an HPCIA, but it should be noted that the latter also exhibit high resistance to IP (Human-only). The distinct resistance profiles observed between *E. faecalis* and *E. faecium* underscore the importance of species-level identification in AMR surveillance. Ecological specialisation and differences in genome plasticity are likely to influence the capacity of these species to acquire, maintain, and disseminate resistance determinants. In particular, the recognised association of *E. faecium* with healthcare-associated infections raises concerns regarding the broader circulation of resistance genes across animal, environmental, and human reservoirs, even when direct transmission pathways cannot be demonstrated [30,31].

Both *E. coli* and enterococci possess characteristics that facilitate their persistence beyond the host environment. Their capacity to survive in manure-amended soils, sediments, and aquatic environments increases opportunities for interaction with environmental microbiota and promotes the exchange of resistance determinants through horizontal gene transfer [27,34,35]. Recent One Health studies have demonstrated genetic overlap among livestock-associated, environmental, and clinical bacterial populations, supporting the concept that environmental compartments function not only as recipients of resistant bacteria but also as active reservoirs where resistance determinants can be maintained, amplified, and redistributed [27,34,35,36]. From this perspective, the environmental dimension of AMR should not be viewed solely as a consequence of livestock production but also as a component that may contribute to the long-term persistence and circulation of resistance within interconnected ecosystems.

This study has several limitations that should be considered when interpreting the results. First, its cross-sectional design provides a snapshot of antimicrobial resistance patterns and does not allow assessment of temporal trends or causal relationships between antimicrobial exposure and resistance. Second, information on antimicrobial use at the farm level was not available, preventing direct evaluation of associations between antimicrobial consumption and resistance prevalence. Third, the study relied on phenotypic susceptibility testing, and molecular characterization of resistance determinants and genetic relatedness was not performed. Consequently, the mechanisms underlying the observed resistance profiles and the potential contribution of mobile genetic elements could not be assessed. Nevertheless, the study provides valuable baseline data on resistance to WHO-priority antimicrobials in Portuguese pig production and contributes to ongoing antimicrobial resistance surveillance efforts within a One Health framework.

## 5. Conclusions

This study demonstrates a high burden of antimicrobial resistance frequency, calculated at the isolate level, in commensal *E. coli* and *Enterococcus* isolated from piglets in Portugal, focusing on antimicrobials of high and critical importance for human medicine and contextualising resistance profiles using WHO priority classifications [13]. Resistance to these antimicrobials was common, particularly ciprofloxacin (43.2% in *E. coli* and 45.0% in *E. faecalis*); resistance to third- and fourth-generation cephalosporins was also identified in *E. coli* isolates, and multidrug resistance was detected in 61.2% of these isolates. *E. faecium* showed a notably high prevalence of imipenem resistance (64.4%).

Although this study does not assess transmission pathways, it demonstrates that resistant bacteria are present at an early stage of pig production. These findings support the continued strengthening of antimicrobial stewardship practices and the inclusion of piglets in integrated One Health antimicrobial resistance surveillance programs in Portugal, particularly for monitoring resistance to critically important antimicrobials and multidrug-resistant bacteria, and improved manure and environmental management strategies to mitigate the persistence and dissemination of antimicrobial resistance across ecosystems.

## Figures and Tables

**Table 1:** Primers and PCR conditions used in identification of *Enterococcus* species.

Species	Primer Sequence	PCR Conditions	Amplicon Size (bp)	Reference
*Enterococcus* spp.	TCA ACC GGG GAG GGT	95 °C, 10 s; 60 °C, 5 s; 72 °C, 29 s	733	[15]
	ATT ACT AGC GAT TCC GG			
*E. faecalis*	ATC AAG TAC AGT TAG TCT	95 °C, 10 s; 44 °C, 5 s; 72 °C, 38 s	941	[15]
	ACG ATT CAA AGC TAA CTG			
*E. faecium*	TTG AGG CAG ACC AGA TTG ACG	95 °C, 10 s; 55 °C, 5 s; 72 °C, 26 s	658	[15]
	TAT GAC AGC GAC TCC GAT TCC			

## Data Availability

The original contributions presented in this study are included in the article/Supplementary Materials. Further inquiries can be directed to the corresponding author.

## Supplementary Materials

The following supporting information can be downloaded at: https://www.mdpi.com/article/10.3390/microorganisms14102342/s1, Table S1: Susceptibility profiles of *Escherichia coli*; Table S2: Susceptibility profiles of enterococci.
