# Supplementary material for: Antimicrobial Resistance to WHO-Categorised Critically Important Antimicrobials in Piglet-Derived Escherichia coli and Enterococci: A One Health Approach
Source: Microorganisms. 2026 Oct 14;14(10):2342. doi: 10.3390/microorganisms14102342 (PMC13707596; doi:10.3390/microorganisms14102342)
Supplement: Supplementary file 1 [file microorganisms-14-02342-s001.zip › microorganisms-4459143-supplementary.pdf]

Table S1. Susceptibility profiles of *Escherichia coli*

Bacteria Id	IP	AMK	CIP	SXT	AZT	FEP	CAZ	AMC	PIP	FOX
1.1	S	S	R	R	S	S	S	S	S	S
1.2	S	S	R	S	S	S	S	S	S	S
1.3	S	S	R	S	S	S	S	S	R	S
1.4	S	S	S	R	S	S	S	S	S	S
2.1	S	S	S	S	S	S	S	S	S	S
2.2	S	S	R	R	S	S	S	S	S	S
2.3	S	S	R	S	S	S	S	R	S	S
2.4	S	S	R	R	S	S	S	R	S	S
3.1	S	S	S	S	S	S	S	S	S	S
3.2	S	S	S	S	S	S	S	S	S	S
3.3	S	S	R	R	S	S	S	R	R	S
3.4	S	S	R	R	S	S	S	R	R	S
4.1	S	S	S	S	S	S	S	S	R	S
4.2	S	S	S	S	S	S	S	R	R	S
4.3	S	S	R	S	S	S	S	S	R	S
4.4	S	S	S	S	S	S	S	S	R	S
5.1	S	S	R	R	S	S	S	R	S	S
5.2	S	S	R	R	S	S	S	R	S	S
5.3	S	S	R	R	S	S	S	S	S	S
5.4	S	S	R	R	S	S	S	R	S	S
6.1	S	S	R	R	R	S	R	S	R	S
6.2	S	S	R	R	R	R	R	S	R	S
6.3	S	S	R	R	R	R	R	S	R	S
6.4	S	S	R	S	R	R	R	R	R	S
7.1	S	S	R	R	S	S	S	S	R	R
7.2	S	S	S	R	S	R	S	S	R	R
7.3	S	S	R	R	S	S	S	S	R	R
7.4	S	S	R	R	S	S	S	S	R	R
8.1	S	S	R	S	R	S	R	R	R	S
8.2	S	S	R	R	R	R	R	R	R	S
8.3	S	S	R	S	R	S	R	R	R	S
8.4	S	S	S	R	R	R	S	S	R	R
9.1	S	S	S	R	R	R	S	S	R	S
9.2	S	S	S	S	R	R	S	S	R	S
9.3	S	S	R	S	R	R	S	S	R	S
9.4	S	S	R	S	R	R	S	S	R	S
9.5	S	S	R	S	R	S	R	R	R	R
10.1	S	S	R	R	R	S	R	R	R	R
10.2	S	S	S	R	S	S	S	S	R	S
10.3	S	S	R	S	R	S	R	R	R	R

10.4	S	S	R	R	R	R	R	S	R	S
11.1	S	S	S	R	S	S	S	S	R	S
11.2	S	S	S	R	S	S	S	S	R	S
11.3	S	S	R	R	S	S	S	S	R	S
11.4	S	S	S	S	S	S	S	S	R	S
12.1	S	S	S	S	S	S	S	R	R	S
12.2	S	S	S	S	S	S	S	S	R	S
12.3	S	S	S	S	S	S	S	S	R	R
12.4	S	S	S	S	S	S	S	S	R	S
13.1	S	S	S	S	S	S	S	S	R	S
13.2	S	S	S	R	S	S	S	R	R	S
13.3	S	S	S	R	S	S	S	S	R	S
13.4	S	S	S	S	S	S	S	S	R	S
14.1	S	S	S	S	S	S	S	S	R	S
14.2	S	S	S	S	S	S	S	R	R	S
14.3	S	S	S	S	S	S	S	S	R	S
14.4	S	S	S	S	S	S	S	S	R	S
15.1	S	S	S	S	S	S	S	S	R	S
15.2	S	S	S	S	S	S	S	S	R	S
15.3	S	S	R	R	S	S	S	S	R	S
15.4	S	S	S	S	S	S	S	S	R	S
16.1	S	S	S	S	R	R	S	S	R	S
16.2	S	S	S	S	R	R	S	S	R	S
16.3	S	S	S	S	R	R	S	R	R	S
16.4	S	R	S	S	R	R	S	S	R	S
17.1	S	S	S	S	R	R	S	S	R	S
17.3	S	S	S	S	R	R	S	S	R	S
17.4	S	S	S	S	S	S	S	S	S	S
18.1	S	S	S	R	S	S	S	S	R	S
18.2	S	S	S	S	S	S	S	S	S	S
18.4	S	S	S	S	S	S	S	S	S	S
19.1	S	S	S	S	R	R	S	S	R	S
19.2	S	S	S	S	R	R	S	S	R	S
19.3	S	S	S	S	R	R	S	S	R	S
19.4	S	S	S	S	R	R	S	S	R	S
20.1	S	S	R	R	R	S	S	S	R	S
20.2	S	S	R	R	S	S	S	S	R	S
20.3	S	S	R	R	S	S	S	R	R	S
20.4	S	S	S	R	S	S	S	S	R	S
21.1	S	S	S	R	S	S	S	S	R	S
21.2	S	S	S	R	S	S	S	S	R	S
21.3	S	S	S	R	S	S	S	R	R	S
21.4	S	S	R	R	S	S	S	S	R	S
22.1	S	S	S	R	S	S	S	S	R	S

22.2	S	S	R	S	S	S	S	S	R	S
22.3	S	S	S	S	S	S	S	S	R	S
22.4	S	S	S	S	S	S	S	S	R	S
23.1	S	S	S	R	R	R	R	R	R	S
23.2	S	S	S	R	R	R	R	R	R	S
23.3	S	S	S	R	R	R	R	S	R	S
23.4	S	S	S	R	R	R	R	S	R	S
24.1	S	S	S	R	R	R	S	R	R	S
24.2	S	S	R	S	S	S	S	S	R	S
24.3	S	S	S	R	R	S	S	S	R	S
24.4	S	S	R	R	S	S	S	S	R	S
25.1	S	S	S	S	S	S	S	S	S	S
25.2	S	S	S	S	S	S	S	S	S	S
25.3	S	S	S	R	R	R	S	R	R	S
25.4	S	S	S	R	S	S	S	S	R	S
26.1	S	S	S	R	S	S	S	S	R	S
26.2	S	S	S	R	S	S	S	S	R	S
26.3	S	S	S	R	S	S	S	R	R	S
26.4	S	S	S	R	R	R	S	S	R	S
27.1	S	S	S	R	R	R	S	S	R	S
27.2	S	S	S	R	R	R	S	R	R	S
27.3	S	S	R	R	R	R	S	R	R	S
27.4	S	S	S	R	S	S	S	R	R	S
28.1	S	S	R	R	R	R	S	S	R	S
28.2	S	S	S	R	S	R	S	S	R	S
28.3	S	S	R	R	S	S	S	S	R	S
28.4	S	S	S	R	S	S	S	S	R	S
29.1	S	S	S	R	R	R	S	S	R	S
29.2	S	S	S	R	R	R	S	S	R	S
29.3	S	S	S	R	S	S	S	S	R	S
29.4	S	S	S	R	S	S	S	S	R	S
30.1	S	S	S	S	S	S	S	S	S	S
30.2	S	S	S	S	S	S	S	S	S	S
30.3	S	S	S	R	S	S	S	S	R	S
30.4	S	S	S	S	S	S	S	S	S	S
31.1	S	S	S	R	S	S	S	S	R	S
31.2	S	S	S	R	S	S	S	S	R	S
31.3	S	S	S	S	R	R	R	R	R	R
31.4	S	S	S	S	S	S	S	S	S	S
32.1	S	S	R	S	S	S	S	S	R	S
32.2	S	S	R	S	S	S	S	S	R	R
32.3	S	S	S	S	S	S	S	S	S	S
32.4	S	S	R	S	S	S	S	S	R	S
33.1	S	S	S	S	S	S	S	S	S	S

33.2	S	S	S	S	S	S	S	S	S	S
33.3	S	S	R	S	S	S	S	R	R	S
33.4	S	S	S	R	S	R	S	S	S	S
34.1	S	S	R	S	S	S	S	R	R	S
34.2	S	S	S	R	S	S	S	R	R	S
34.3	S	S	S	R	S	S	S	R	R	S
34.4	S	S	S	S	S	S	S	R	S	S
35.1	S	S	S	R	S	S	S	R	R	S
35.3	S	S	S	S	S	S	S	S	S	S
35.4	S	S	S	R	S	S	S	R	R	S
36.1	S	S	R	S	R	R	R	R	R	R
36.2	S	S	R	R	R	S	S	S	R	S
36.3	S	R	R	R	S	S	S	R	R	S
36.4	S	S	R	R	S	S	S	R	R	S
36.5	S	S	R	R	S	S	S	R	R	S
36.6	S	S	S	S	S	S	S	R	S	S
37.2	S	S	S	S	S	S	S	S	S	S
37.3	S	S	S	R	S	S	S	S	R	S
37.4	S	S	S	S	S	R	S	S	R	S
37.5	S	S	R	R	S	S	S	S	R	S
37.6	S	S	S	S	S	R	S	S	R	S
38.1	S	S	R	S	S	S	S	S	R	S
38.2	S	S	R	R	S	S	S	S	R	S
38.3	S	S	S	R	S	S	S	S	R	S
38.4	S	S	R	R	S	S	S	R	R	S
38.5	S	S	S	S	S	S	S	S	S	S
38.6	S	S	R	R	S	S	S	R	R	S
39.1	S	S	S	S	R	S	R	R	R	R
39.2	S	S	S	S	R	S	R	R	R	R
39.3	S	S	S	R	S	S	S	S	S	S
39.5	S	S	R	R	R	S	R	R	R	R
40.1	S	S	R	S	S	S	S	R	R	S
40.2	S	S	S	R	S	S	S	R	S	S
40.3	S	S	S	S	S	S	S	R	S	S
40.4	S	S	R	R	S	S	S	S	R	S
40.5	S	S	R	R	S	S	S	R	R	S
40.6	S	S	S	R	S	S	S	R	R	S
41.1	S	S	R	R	S	S	S	R	R	S
41.2	S	S	R	R	S	S	S	R	R	S
41.3	S	S	R	R	S	S	S	R	R	S
41.4	S	S	R	R	S	S	S	R	R	S
41.5	S	S	R	R	S	S	S	R	R	S
41.6	S	S	R	R	S	S	S	R	R	S
42.3	S	S	R	R	S	S	S	R	R	S

42.4	S	S	R	R	S	S	S	R	R	S
42.5	S	S	R	R	S	S	S	R	R	S
42.6	S	S	R	R	S	S	S	R	R	S
43.1	S	R	S	S	S	S	S	R	R	S
43.2	S	S	S	R	S	S	S	R	S	S
43.3	S	S	S	R	S	S	S	R	R	S
43.4	S	S	R	R	S	S	S	R	R	S
43.5	S	S	R	R	S	S	S	R	R	S
43.6	S	R	S	R	S	S	S	R	R	S
44.1	S	R	R	R	S	S	S	R	S	S
44.2	S	R	R	R	S	S	S	R	R	S
44.3	S	S	R	R	S	S	S	R	R	S
44.4	S	S	R	R	S	S	S	R	R	S
45.1	S	R	R	R	S	S	S	R	R	S
45.2	S	S	S	R	S	S	S	R	S	S
45.3	S	R	R	R	S	S	S	R	R	S
45.4	S	S	R	S	S	S	S	R	R	S
45.5	S	S	S	S	S	S	S	S	S	S
45.6	S	S	S	S	S	S	S	R	S	S
46.1	S	S	S	S	S	S	S	R	S	S
46.2	S	S	S	S	S	S	S	R	S	S
46.3	S	S	S	S	S	S	S	R	S	S
46.4	S	S	S	S	S	S	S	R	S	S
46.5	S	S	S	S	S	S	S	R	S	S
46.6	S	S	S	S	S	S	S	R	S	S
47.1	S	R	R	R	S	S	S	R	S	S
47.2	S	S	R	R	S	S	S	S	S	S
47.3	S	S	R	R	S	S	S	R	R	S
47.4	S	R	R	R	S	S	S	R	R	S
47.5	S	R	R	R	S	S	S	R	S	S
47.6	S	R	R	R	S	S	S	R	R	S
48.1	S	R	S	S	S	S	S	R	R	S
48.2	S	R	S	R	S	S	S	R	R	S
48.3	S	R	S	S	S	S	S	R	S	S
48.4	S	S	S	R	S	S	S	R	R	S
48.5	S	S	R	R	R	R	R	R	R	S
48.6	S	R	S	S	S	S	S	R	R	S
49.1	S	R	R	R	R	R	R	R	R	S
49.2	S	R	R	R	S	S	S	R	R	R
49.3	S	R	S	R	S	R	S	R	R	R
49.4	S	R	R	R	S	S	S	R	R	R
49.5	S	R	R	R	S	S	S	R	R	R
50.1	S	R	R	R	S	S	S	R	R	S
50.2	S	R	S	R	S	S	S	R	R	R

50.3	S	R	S	S	S	S	S	R	S	S
50.4	S	R	S	S	S	S	S	R	R	R
50.5	S	R	R	R	S	S	S	R	R	R
51.1	S	R	R	R	S	S	S	R	R	S
51.2	S	S	R	R	S	S	S	R	R	S
51.3	S	R	R	R	S	S	S	R	R	S
51.4	S	R	R	R	S	S	S	R	R	S
51.5	S	R	R	R	S	S	S	R	R	S
52.1	S	R	R	R	R	R	R	R	R	S
52.2	S	R	R	R	R	R	S	R	R	S
52.3	S	R	R	R	S	S	S	R	R	S
52.4	S	S	R	R	R	R	R	R	R	S
52.5	S	R	R	R	S	S	S	R	R	R
53.1	S	S	R	R	S	S	S	R	R	R
53.2	S	R	R	R	R	R	R	R	R	S
53.3	S	R	R	R	S	S	R	R	R	S
53.4	S	R	R	R	R	R	R	R	R	S
53.5	S	R	R	R	R	R	R	R	R	R
54.1	S	R	R	R	S	S	S	R	R	R
54.2	S	R	R	S	S	S	S	R	S	S
54.3	S	R	R	R	S	S	S	R	R	S
54.4	S	R	R	R	S	S	S	R	R	R
54.5	S	R	R	R	S	S	S	R	R	S
55.1	S	R	S	S	S	S	S	R	R	S
55.3	S	R	R	S	S	S	S	R	S	S
55.5	S	R	R	R	R	R	R	R	R	R
56.1	S	R	R	R	S	S	S	R	R	R
56.3	S	R	R	R	S	S	S	R	R	S
56.4	S	R	R	R	S	S	S	R	R	S
56.5	S	R	R	R	S	S	R	R	R	S
56.6	S	R	R	R	S	S	S	R	R	S
57.1	S	R	S	R	R	R	S	R	S	S
57.4	S	R	S	S	R	R	S	R	S	S
57.5	S	R	S	R	S	S	S	R	S	S
57.6	S	R	S	S	S	R	R	R	S	S
58.1	S	R	R	S	S	S	S	R	S	S
58.2	S	R	S	S	S	S	S	R	S	S
58.3	S	R	S	S	S	S	S	R	S	S
58.4	S	R	S	S	S	S	S	R	S	S
58.5	S	R	S	S	S	S	S	R	S	S
58.6	S	R	S	S	S	S	S	R	S	S
59.1	S	R	S	S	S	S	S	R	S	S
59.2	S	R	S	S	S	S	S	R	S	S
59.3	S	R	S	S	S	S	S	R	S	S

59.4	S	R	S	S	S	S	S	R	S	S
59.5	S	R	S	S	S	S	S	R	S	S
59.6	S	R	S	S	S	S	S	R	S	S
60.1	S	R	S	R	S	S	S	R	S	S
60.2	S	S	S	R	S	S	S	R	R	S
60.3	S	S	S	R	S	S	S	R	S	S
60.4	S	R	R	S	S	S	S	R	S	S
60.5	S	R	R	R	S	S	S	R	R	S
60.6	S	R	R	S	S	S	S	R	R	R
61.4	S	S	R	S	S	S	S	R	R	R
62.1	S	S	S	S	S	S	S	R	S	S
62.2	S	R	R	R	S	S	S	R	R	S
62.3	S	R	R	R	S	S	S	R	S	S
62.4	S	R	R	R	S	S	S	R	R	S
62.5	S	R	R	R	S	S	S	R	R	S
62.6	S	R	S	S	S	S	S	R	S	S
63.1	S	R	R	R	S	S	R	R	R	S
63.2	S	R	R	R	S	S	S	R	R	S
63.3	S	S	R	R	S	S	S	R	R	S
63.4	S	S	R	R	S	S	S	R	R	S
63.5	S	R	R	R	S	S	S	R	R	R
63.6	S	R	R	R	S	S	S	R	R	S
64.1	S	R	S	S	S	S	S	R	S	S
64.2	S	R	S	S	S	S	S	R	S	S
64.3	S	R	S	S	S	S	S	R	S	S
64.4	S	R	S	S	S	S	S	R	S	S
64.5	S	R	S	S	S	S	S	R	S	S
64.6	S	S	S	S	S	S	S	R	S	S
65.2	S	R	S	S	S	S	S	R	S	S
65.4	S	R	S	S	S	S	S	R	S	S
65.5	S	R	S	S	S	S	S	R	S	S
65.6	S	S	S	S	S	S	S	R	S	S
66.1	S	R	S	R	S	S	S	R	S	S
66.2	S	R	S	R	S	S	S	R	S	S
66.3	S	S	S	R	S	S	S	R	S	S
66.4	S	R	S	R	S	S	S	R	S	S
66.5	S	S	S	R	S	S	S	R	S	S
66.6	S	S	S	R	S	S	S	R	S	S
67.1	S	R	S	S	S	S	S	R	R	S
67.2	S	R	S	S	S	S	S	R	R	R
67.3	S	R	S	S	S	S	S	R	R	R
67.4	S	S	S	S	S	S	S	R	R	S
67.5	S	R	S	S	S	S	S	R	R	S
67.6	S	R	S	S	S	S	S	R	R	S

68.3	S	R	R	S	R	R	R	R	R	S
68.5	S	S	R	S	S	S	S	R	R	S
69.2	S	S	S	S	S	S	S	R	S	S
70.1	S	S	S	S	S	S	S	R	R	S
70.2	S	R	S	R	S	S	S	R	R	S
70.3	S	S	R	R	S	S	S	R	R	S
70.4	S	R	R	R	S	S	S	R	R	S
71.1	S	S	R	R	S	S	S	R	S	S
71.2	S	R	R	R	R	R	R	R	R	R
71.3	S	S	R	S	R	S	R	R	R	R
71.4	S	R	R	S	R	R	R	R	R	R
71.5	S	R	R	S	S	S	S	R	R	R
71.6	S	R	S	S	S	S	S	R	S	S
72.1	S	S	S	S	S	S	S	R	S	S
72.2	S	S	S	R	S	S	S	R	R	S
72.3	S	S	S	S	S	S	S	R	S	S
72.4	S	S	S	S	S	S	S	R	S	S
72.5	S	R	R	S	S	S	S	R	S	R
72.6	S	S	S	S	S	S	S	R	S	S
73.1	R	R	S	R	S	S	S	R	S	S
73.2	S	R	S	R	S	S	S	R	S	S
73.3	R	R	S	R	S	S	S	R	S	S
73.4	S	R	S	R	S	S	S	R	S	S
73.5	S	R	S	R	S	S	S	R	S	S
74.1	R	S	R	R	R	R	R	R	R	R
74.2	S	S	S	S	S	S	S	R	S	S
74.3	R	R	S	R	S	R	R	R	R	S
74.4	R	R	R	R	S	S	S	R	R	R
74.5	R	R	S	R	S	S	S	R	S	S
74.6	S	R	S	S	S	S	S	R	S	S
75.1	R	R	S	R	S	S	S	R	S	S
75.2	S	S	S	S	S	S	S	R	S	S
75.3	R	R	R	R	R	R	R	R	R	R
75.4	S	S	S	S	S	S	S	R	S	S
75.5	S	S	S	S	S	S	S	R	S	S
75.6	R	S	S	S	S	S	S	R	S	S

S, susceptible; R, resistant; IP, Imipenem; AMK, Amikacin; CIP, Ciprofloxacin; SXT, Sulfomethoxazol+trimethoprin; AZT, Aztreonam; FEP, Cefepim; CAZ, ceftazidime; AMC, amoxicillin+clavulanic acid; PIP, Piperacillin; FOX, cefoxitin

Table S2. Susceptibility profiles of enterococci

Bacteria Id	Species	VAN	CIP	LNZ	TIG	AMP	IP
3.6	*E. faecium*	S	S	S	S	S	S
3.8	*Enterococcus* spp.	S	S	S	S	S	S
4.1	*E. faecalis*	S	S	S	S	S	R
4.2	*E. faecalis*	S	S	S	S	S	R
5.5	*E. faecium*	S	S	S	S	S	S
5.7	*Enterococcus* spp.	S	S	S	S	S	S
7.4	*E. faecalis*	S	S	S	S	S	S
8.1	*E. faecium*	S	S	S	S	S	S
8.2	*Enterococcus* spp.	R	R	R	S	S	S
8.3	*E. faecalis*	S	S	S	S	S	S
8.4	*E. faecalis*	S	S	S	S	S	R
8.5	*Enterococcus* spp.	S	S	S	S	S	R
8.6	*Enterococcus* spp.	R	R	S	S	S	S
9.2	*E. faecalis*	S	R	S	S	S	S
9.3	*E. faecalis*	R	R	S	S	R	S
9.4	*Enterococcus* spp.	R	R	S	S	R	S
10.1	*E. faecalis*	S	R	S	S	S	S
12.3	*Enterococcus* spp.	S	R	S	S	S	S
16.2	*E. faecium*	S	S	S	S	S	R
16.3	*Enterococcus* spp.	S	R	S	S	S	S
16.4	*E. faecium*	S	S	S	S	S	R
17.2	*Enterococcus* spp.	S	S	S	S	S	S
18.6	*Enterococcus* spp.	S	S	S	S	S	S
21.3	*E. faecalis*	S	S	S	S	S	S
22.2	*E. faecium*	S	S	S	S	S	S
22.3	*Enterococcus* spp.	S	S	S	S	S	R
22.4	*E. faecium*	S	S	S	S	S	R
22.5	*E. faecium*	S	S	S	S	S	S
22.6	*E. faecium*	S	S	S	S	S	S
22.7	*Enterococcus* spp.	S	S	S	S	S	R
23.1	*E. faecium*	S	S	S	S	S	R
23.2	*E. faecium*	S	S	S	S	S	R
23.3	*E. faecium*	S	S	S	S	S	R
23.4	*Enterococcus* spp.	S	S	S	S	S	R
24.1	*Enterococcus* spp.	S	S	S	S	S	R
24.2	*E. faecium*	S	S	S	S	S	R
25.1	*E. faecium*	S	R	S	S	S	S
25.3	*Enterococcus* spp.	S	R	S	S	S	S
25.4	*Enterococcus* spp.	S	R	S	S	S	S
26.3	*E. faecium*	S	S	S	S	S	R
27.1	*E. faecalis*	S	R	S	S	S	S
27.2	*E. faecalis*	S	S	S	S	S	S
27.3	*E. faecium*	S	S	S	S	S	R

27.4	*E. faecium*	S	S	S	S	S	R
31.1	*E. faecalis*	S	S	S	S	S	S
31.2	*E. faecalis*	S	S	S	S	S	S
31.3	*Enterococcus* spp.	S	S	S	S	S	S
31.5	*E. faecium*	S	S	S	S	R	R
31.6	*E. faecalis*	S	R	S	S	S	S
31.7	*E. faecalis*	S	R	S	S	S	S
31.8	*E. faecalis*	S	R	S	S	S	S
31.9	*E. faecium*	S	R	S	S	S	R
32.1	*E. faecalis*	S	S	S	S	S	S
32.2	*E. faecalis*	S	R	S	S	S	S
32.3	*E. faecalis*	S	S	S	S	S	S
32.4	*E. faecalis*	S	S	S	S	S	S
34.1	*E. faecium*	S	S	S	S	S	S
34.3	*E. faecalis*	S	S	S	S	S	S
34.4	*E. faecalis*	S	R	S	S	S	S
35.1	*E. faecium*	S	R	S	S	S	S
35.2	*E. faecalis*	S	R	S	S	S	S
35.3	*E. faecalis*	S	R	S	S	S	S
35.4	*E. faecalis*	S	R	S	S	S	S
36.8	*E. faecium*	S	S	S	S	S	S
38.1	*Enterococcus* spp.	S	S	S	S	S	S
38.2	*Enterococcus* spp.	S	S	S	S	S	S
41.4	*Enterococcus* spp.	S	R	S	S	S	S
41.7	*Enterococcus* spp.	S	R	S	S	S	S
41.8	*Enterococcus* spp.	S	R	S	S	S	S
42.1	*Enterococcus* spp.	S	S	S	S	S	S
42.2	*Enterococcus* spp.	S	S	S	S	S	S
42.3	*E. faecium*	S	S	S	S	S	R
42.4	*E. faecium*	S	S	S	S	S	R
42.5	*Enterococcus* spp.	S	S	S	S	S	R
42.6	*Enterococcus* spp.	S	S	S	S	S	S
42.7	*Enterococcus* spp.	S	S	S	S	S	S
43.3	*Enterococcus* spp.	S	R	S	S	S	S
43.4	*E. faecium*	S	S	S	S	S	R
44.1	*Enterococcus* spp.	S	S	S	S	S	S
44.2	*E. faecium*	S	S	S	S	S	R
44.3	*E. faecium*	S	S	S	S	S	S
44.4	*E. faecium*	S	S	S	S	S	S
44.5	*E. faecalis*	S	S	S	S	S	S
44.6	*E. faecium*	S	R	S	S	S	R
44.7	*E. faecium*	S	S	S	S	S	R
44.8	*E. faecalis*	S	S	S	S	S	S
46.2	*E. faecalis*	S	S	S	S	S	S
46.3	*Enterococcus* spp.	S	S	S	S	S	S

46.5	*Enterococcus* spp.	S	S	S	S	S	S
46.6	*Enterococcus* spp.	S	S	S	S	S	S
47.2	*Enterococcus* spp.	S	R	S	S	S	S
47.3	*Enterococcus* spp.	S	R	S	S	S	S
47.4	*Enterococcus* spp.	S	R	S	S	S	S
47.5	*Enterococcus* spp.	S	R	S	S	S	S
47.6	*Enterococcus* spp.	S	R	S	S	S	S
48.1	*E. faecium*	S	S	S	S	S	R
48.2	*E. faecium*	S	S	S	S	S	R
50.1	*E. faecium*	S	R	S	S	S	R
50.3	*E. faecalis*	S	S	S	S	S	R
55.2	*Enterococcus* spp.	S	S	S	S	S	S
56.1	*E. faecalis*	S	S	S	S	S	S
56.4	*E. faecalis*	S	S	S	S	S	S
57.1	*Enterococcus* spp.	S	S	S	S	S	S
57.3	*Enterococcus* spp.	S	S	S	S	S	S
57.5	*E. faecium*	S	S	S	S	S	S
57.6	*E. faecium*	S	S	S	S	S	S
59.1	*E. faecium*	S	S	S	S	S	S
60.1	*E. faecalis*	S	R	S	S	S	S
60.2	*Enterococcus* spp.	S	R	S	S	S	S
60.3	*Enterococcus* spp.	S	R	S	S	S	S
60.4	*Enterococcus* spp.	S	R	S	S	S	S
60.5	*Enterococcus* spp.	S	R	S	S	S	S
60.6	*E. faecalis*	S	R	S	S	S	S
60.7	*E. faecalis*	S	R	S	S	S	S
62.1	*E. faecalis*	S	S	S	S	S	R
62.2	*E. faecalis*	S	S	S	S	S	R
63.5	*E. faecium*	S	S	S	S	S	R
66.3	*E. faecium*	S	S	S	S	S	S
72.1	*E. faecium*	S	S	S	S	S	R
72.5	*E. faecalis*	S	R	S	S	S	S
72.6	*E. faecium*	R	R	S	S	S	R
73.5	*E. faecium*	S	R	R	S	S	R
73.6	*E. faecalis*	S	R	S	S	S	S
73.7	*E. faecalis*	S	R	S	S	S	S
74.1	*E. faecium*	S	S	S	S	S	R
74.2	*E. faecalis*	S	S	S	S	S	S
74.3	*Enterococcus* spp.	S	S	S	S	S	S
74.5	*E. faecium*	S	S	S	S	S	R
74.6	*E. faecium*	S	S	S	S	R	R
75.3	*E. faecium*	S	S	S	S	S	R

S, susceptible; R, resistant; VAN, vancomycin; CIP, Ciprofloxacin; LNZ, linezolid; TIG, tigecycline; AMC, amoxicillin+clavulanic acid; IP, Imipenem
